# The medical schools outcomes database project: Australian medical student characteristics

**DOI:** 10.1186/1472-6920-14-180

**Published:** 2014-08-29

**Authors:** Baldeep Kaur, Angela Carberry, Nathaniel Hogan, Don Roberton, Justin Beilby

**Affiliations:** Medical Deans Australia and New Zealand Inc. St James Campus (C13), University of Sydney, Level 6, 173-175 Phillip Street, 2000 Sydney, NSW Australia; Faculty of Health Sciences, University of Adelaide, Adelaide, SA Australia

**Keywords:** Medical education, Medical workforce, Health, Medical student, Medical doctors, Longitudinal, Medicine, Methodology, Interns, Internship

## Abstract

**Background:**

Global medical workforce requirements highlight the need for effective workforce planning, with the overall aims being to alleviate doctor shortages and prevent maldistribution. The Medical Schools Outcomes Database and Longitudinal Tracking (MSOD) Project provides a foundation for evaluating outcomes of medical education programs against specified workforce objectives (including rural and areas of workforce needs), assisting in medical workforce planning, and provision of a national research resource. This paper describes the methodology and baseline results for the MSOD project.

**Methods:**

The MSOD Project is a prospective longitudinal multiple-cohort study. The project invites all commencing and completing Australian medical students to complete short questionnaires. Participants are then asked to participate in four follow-up surveys at 1, 3, 5 and 8 years after graduation.

**Results:**

Since 2005, 30,635 responses for medical students (22,126 commencing students and 8,509 completing students) in Australia have been collected. To date, overall eligible cohort response rates are 91% for commencing students, and 83% for completing students. Eighty three percent of completing medical student respondents had also completed a commencing questionnaire.

Approximately 80% of medical students at Australian medical schools are Australian citizens. Australian medical schools have only small proportions of Indigenous students. One third of medical students speak a language other than English at home.The top three vocational choices for commencing medical students were surgery, paediatrics and child health and general practice. The top three vocational choices for completing students were surgery, adult medicine/ physician, and general practice. Overall, 75.7% of medical students changed their first career preference from commencement to exit from medical school.

Most commencing and completing medical students wish to have their future medical practice in capital cities or in major urban centers. Only 8.1% of commencing students and 4.6% of completing students stated an intention to have their future medical practice in smaller towns and small communities.

**Conclusions:**

The MSOD longitudinal project is now an established national resource that is beginning to generate significant research outputs, along with providing key information for workforce planning and policy makers. The project has now expanded to enrol New Zealand medical students.

## Background

Globally there is a well-recognised shortage of healthcare workers, highlighting the need for effective health workforce planning [1–8]. There are relatively few key medical workforce longitudinal studies worldwide that have aimed to purposefully and proactively inform national workforce planning. Some notable longitudinal studies are the BMA Cohort Doctor study [9], the UK Medical Careers Research Group (UKMCRG) studies [10], the Young Doctor Cohort of the Longitudinal Study of Norwegian Medical Students and Doctors (NORDOC) [11], the Jefferson Longitudinal study of Medical Education [12], and the Medicine in Australia: Balancing Employment and Life (MABEL) longitudinal surveys of medical graduates in practice [13].

To facilitate and prioritise medical workforce planning in Australia, the Australian government formed Health Workforce Australia (HWA) [14]. Similarly, the New Zealand government established Health Workforce New Zealand (HWNZ) to provide leadership, co-ordination and oversight of planning and development of the health workforce across New Zealand’s health and disability sector [15].

Responding to this need, and recognising that this requirement for data collection and analysis was a key planning workforce priority, Medical Deans Australia and New Zealand Inc. (Medical Deans Inc.) established the Medical Schools Outcomes Database and Longitudinal Tracking (MSOD) Project in 2005 [14].

The MSOD project objectives [16] include:

-  Provision of an effective, reliable evaluation mechanism for assessing long-term outcomes of educational programs, in particular those aimed at addressing future medical workforce needs (rural health, areas of workforce need, Indigenous health, specialty areas, and others as they arise or are implemented)
-  Provision of a secure, reliable source of accurate, up-to-date data for the purposes of long-term medical workforce planning
-  Determining the effectiveness of targeted programs and interventions in influencing the career decisions of medical students
-  Promotion of strategic reform of medical education policy and programs at the university, state and Commonwealth levels in order to match program and policy frameworks with national health priorities
-  Provision of an information resource for research projects for Australian medical educators that will contribute to the national and international literature on medical education.

The aim of this paper is to describe the methods, and some baseline results, for Australian commencing and completing medical students participating in the MSOD project of Medical Deans Inc.

## Methods

The MSOD project is a longitudinal prospective multiple cohort study of medical students in Australia and New Zealand. Six Australian medical schools participated in the pilot commencing survey in 2005 (see Table 1). From 2006 onwards, the number of participating Australian medical schools (including new medical schools) increased progressively. By 2008, all 18 Australian universities with medical schools had commencing students participating in the data collection. Final year medical students from all Australian medical schools were participating by 2011. The MSOD’s minimum dataset provides the core platform for a wide range of sub-studies and data linkage studies.

**Table 1:** **List of participating Australian and New Zealand medical schools**

Australian Medical Schools	Characteristics
The University of Adelaide	UG
Australian National University	G
Bond University	UG
Deakin University	G
Flinders University	G, P
Griffith University	G, P
James Cook University	UG
The University of Melbourne	UG, G, P
Monash University	UG, G, P
The University of Newcastle/University of New England	UG
The University of New South Wales	UG, P
The University of Notre Dame	G
The University of Queensland	G
The University of Sydney	G, P
University of Tasmania	UG
The University of Western Australia	G, UG
University of Western Sydney	UG
University of Wollongong	G
*University of Auckland*	*New Zealand medical school (UG)*
*University of Otago*	*New Zealand medical school (UG)*

**(G) = Graduate program, (UG) = Undergraduate program (P) Participated in Pilot CMSQ in 2005.**

*Note: Some medical schools have changed from undergraduate to graduate programs during the lifetime of the MSOD project, or have combinations of graduate and undergraduate programs.*

### Study cohorts

Annually, all enrolling medical students at all Australian medical schools, and the two New Zealand medical schools, are invited to participate in the MSOD project, and are then asked to complete a short questionnaire in their commencing, final and selected postgraduate years. A list of these medical schools is shown in Table 1.

### Questionnaire design and questionnaire administration

**Figure 1:**
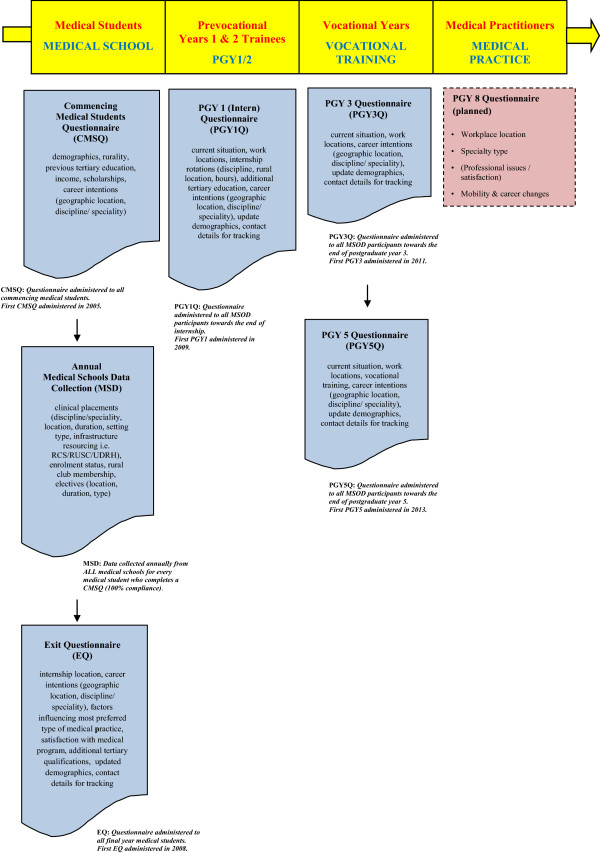
**MSOD Project surveys across the medical education and training continuum (Adapted from Jones, et al.** [18] **).**

Data are collected directly from medical students:

-  upon entry to medical school (Commencing Medical Students Questionnaire, CMSQ);
-  in their final year of medical school (Exit Questionnaire, EQ);

Further questionnaires are being administered, or are being developed, for:

-  one year after completion of their medical studies (PGY1);
-  three years after completion of their medical studies (PGY3);
-  five years after completion of their medical studies (PGY5) and
- eight years after completion of their medical studies (PGY8) (Figure 1).

Further information on individual student placements and electives (Medical Schools Data, MSD) is collected directly from medical schools annually throughout the duration of each student’s program in medical school.

Information from the CMSQ, EQ and MSD will be linked to the postgraduate surveys to provide outcome measures for type and location of medical practice.

The Australian and New Zealand questionnaires differ slightly, taking into account geographical locations and Indigenous status. Further information, including questionnaires, is available on the Project’s website [16].

### CMSQ

The CMSQs are distributed to students within the first few months of commencing medical school. The 17 item questionnaire (35 questions) is paper based due to preference by medical schools for ease of administration to large groups of students at one time. The CMSQ gathers students’ demographics, rural or urban background, enrolment characteristics, previous tertiary education and future career intentions. The majority of questions are quantitative whilst three questions ask for free text responses (relating to scholarship source, previous university qualification and partner occupation).

### MSD

The MSD are collected from each medical school annually for every student who completes the CMSQ via an Excel template.

### EQ

Paper versions of the EQs are distributed to students in their final year of medical school, again as preferred by most medical schools, although a proportion of respondents (34% in 2011) complete an online version of the questionnaire. The 14-item EQ (21 questions) gathers student information about further demographic details, future medical practice intentions (preferred type/vocation of medical practice, and preferred geographic location), factors potentially influencing the choice of future medical practice, and internship placements.

### Postgraduate surveys (PGY1, 3, 5 and 8)

Participants in their post-graduate year one of training (PGY1) are invited to complete the PGY1 questionnaire. The PGY1 questionnaires are completed online by interns at the end of their first year of post-graduate training. A link to the PGY1 questionnaire is emailed to MSOD participants. The PGY1 gathers information on demographics, internship details, future medical practice intentions, factors potentially influencing the choice of future medical practice, and future contact details.

At the end of their third, fifth and eight years after leaving medical school, graduates are being invited to complete PGY3, PGY5 and PGY8 Questionnaires. These questionnaires collect data on demographics, the participant’s current employment and training situation, intentions for future medical practice, factors potentially influencing the choice of future medical practice, and future contact details.

The MSOD Project maintains consistency in each of the surveys with the career intention and other questions across all questionnaires from CMSQ to PGY8, in order to facilitate comparison over the medical education and training continuum.

### Ethics approvals

The study has ongoing approval by the relevant Human Research Ethics Committees (HREC) for each participating medical school. Any research conducted on data contained in the database requires appropriate clearance from a relevant HREC. Further information, including the Project’s Data Access Policy and ethics approval numbers is available on the Project’s website [16].

### Data processes

#### Data dictionary

The Project uses a web-based data dictionary incorporating the definitions and origins of the variables collected through each of the questionnaires and the MSD [16]. The MSOD Data Dictionary provides information to registered users about the MSOD minimum dataset and the rationale for each item in this dataset. It also shows the questions and response values used to collect this information; the individual questionnaires in which these questions are asked; and the database variables to which these responses map.

#### Contacts database

The MSOD Project collects contact details from the respondents via the questionnaires in order to administer subsequent follow up questionnaires with MSOD participants and to keep them informed about the study. These data are stored separately from the participants’ questionnaire data. They are accessible to relevant MSOD staff only.

#### Data management

The completed paper questionnaires (CSMQ and EQ) are returned by the individual medical school to Medical Deans Inc. They are scanned by Educational Assessment Australia (EAA) at the University of New South Wales, Sydney, Australia. Online questionnaires (all surveys) are hosted by the University of Sydney on a secure web server. All data from the scanned paper and online CMSQ, EQ, MSD and postgraduate questionnaires for each survey year are imported into a MySQL database, where they are cleaned and merged into a master file. Data within and between survey years are linked using unique participant identifiers.

The data cleaning process involves importing the data into a *staging* database table; checks that participant identifiers (student ID, gender, date of birth, and medical school) are correct; matches existing participants to their previous responses via a unique identifier; checks missing and multiple response values in scanned data against the original paper responses; and checks text responses to “other” categories to ensure that the response is not identical to one of the response values. A number of derived variables (such as categorisation of occupation, or previous degree type), used for reporting purposes, are then added to the record, and the data are imported into a *production* database table which holds previous years’ data for the particular survey.

A number of measures are taken to ensure data integrity: raw data are kept in CSV and MS Excel (for the MSD) files in a secure network folder, and in archived database tables; all data are backed up on a daily basis and on a weekly basis and archived in a compressed format; and retrospective changes to data are saved into an audit history database table.

Measures are taken to ensure data security: survey data are stored separately from participant identifiers, and participant contact data are also stored separately; a unique key is used to match participants across different surveys and participant contact information; access to MSOD data and to participant information is regulated via a browser-based user interface; MSOD research staff have access to participant survey responses, but not to participant identifiers and contact details, whereas MSOD survey administration staff have access to participant contact information, but not survey responses; and levels of read and write access (whether to survey data or contact information) are defined based on the requirements of staff roles.

#### Relational database

The Project uses a MySQL relational database. A browser-based user interface controls access to the data, and integrates survey administration and collection processes. Simple data queries and data export processes occur through the user interface, and Crystal Reports is used to generate standardised data and survey administration progress reports directly from the database. More complex data analysis is conducted via native SQL, or by exporting data into statistical analysis software, such as SPSS.

#### Data reports

There are two types of data reports produced for the questionnaires – national summary reports, and individual school reports. Information, including data reports by year, is available on the Project’s website [16]. Summary data reports of each of the annual data collections (CMSQ, EQ, and MSD) are provided to the medical schools and are made available to the Commonwealth and the Project’s stakeholder organisations. These reports are de-identified internally and externally, as per MSOD protocols and ethics approvals, to maintain the anonymity of participants.

Data in any public report or publication are presented in forms that do not permit the identification of any individual participant, subgroup or participants from a particular medical school.

Individual school reports, summarising anonymised data responses for participants at that school, are provided to each school along with tables summarising data for all schools combined. This allows each school to view its response profiles compared with the combined response profile for all schools. These individual school reports are provided only to the relevant medical school, and are not available publically.

## Results

In this report, results are presented for the combined commencing, and completing, medical student cohorts from Australian medical schools. New Zealand’s two medical schools also now participate in the MSOD Project, and data analyses for New Zealand schools will be available in the future.

### Participation and response rates

Since 2006 medical students from all Australian medical schools have participated in the CMSQ (15 medical schools in 2006, and this increased to 18 by 2008) (Table 2). As each of these cohorts reached graduation, their respective medical schools administered the EQ, starting with three schools with graduate-entry programs in 2008. In 2011 all 18 Australian universities with medical schools administered the EQ.

**Table 2:** **Responses by Survey and Year**

Year	CMSQ	EQ	
**2005** ^**a**^	878	0	**878**
**2006** ^**b**^	2043	0	**2043**
**2007** ^**c**^	2688	0	**2688**
**2008** ^**d**^	3220	262	**3482**
**2009** ^**d**^	3154	893	**4047**
**2010** ^**d**^	3112	1979	**5091**
**2011** ^**d**^	3560	2553	**6113**
**2012** ^**d**^	3471	2822	**6293**
Total	**22126**	**8509**	30635*

*Data are for Australian Medical Schools only.

^a^6 Australian Medical schools (Pilot Study) participating in CMSQ.

^b^All Australian Universities with Medical Schools (15 schools) participating in CMSQ.

^c^All Australian Universities with Medical Schools (17 schools) participating in CMSQ.

^d^All Australian Universities with Medical Schools (18 schools) participating in CMSQ.

**Figure 2:**
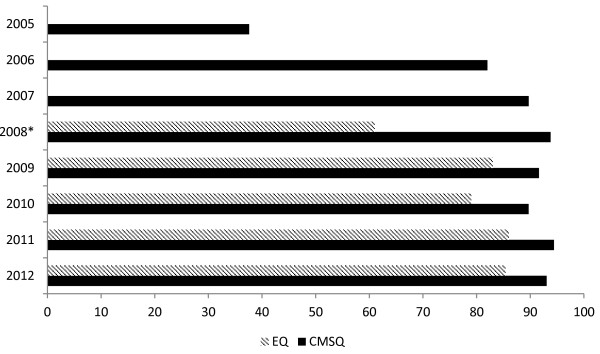
**Eligible cohort response rates by year for CMSQ and EQ.**

At the close of the 2012 surveys, the MSOD project had received 30,635 valid responses (Table 2). Figure 2 shows the eligible cohort response rates (derived as the number of valid responses as a proportion of the total number of students eligible to respond) by year, for the CMSQ and the EQ.

To date the overall eligible cohort response rate is 91% at commencement of medical school, and 83% at completion of medical school. The overall eligible cohort response rate for the most recently completed survey analyses (2012 Survey years) are 93% for the CMSQ, and 85% for the EQ (Figure 2).

Overall, 7,061 (83%) of completing medical student respondents have completed both the CMSQ and EQ.

### Baseline data

#### Commencing medical students

Of the 22,126 commencing students responding to the questionnaires, 47.3% were male and 52.7% were female (12 students did not have responses to this question) (Table 3). Approximately equal proportions of commencing students were participating in direct entry (undergraduate) medical school programs (49.6%), and in graduate programs (50.3%) (Table 3).

**Table 3:** **Socio-demographic characteristics of 30,635 valid responses in the MSOD longitudinal tracking project**

	CMSQ	EQ
	N = 22,126 (%)	N = 8509 (%)
Gender*		
Male	10,462 (47.3)	3723 (45.0)
Female	11,652 (52.7)	4557 (55.0)
Missing/no response	12	229
**Program type**		
Undergraduate	10,983 (49.7)	3,668 (43.2)
Graduate	11,132 (50.3)	4,831 (56.8)
Missing/no response	11	10
**Age (years)**		
<20	11,601 (52.5)	9 (0.1)
21-24	6716 (30.4)	3163 (37.2)
25-29	2549 (11.5)	4057 (47.8)
30-34	722 (3.2)	861 (10.1)
>35	527 (2.4)	409 (4.8)
Missing/no response	11	10
**Citizenship status**		
Australian citizen	17506 (79.4)	6432 (80.6)
New Zealand citizen	599 (2.7)	163 (2.0)
Australian permanent resident	461 (2.1)	108 (1.4)
Temporary entry permit	3366 (15.3)	1248 (15.6)
Other	117 (0.5)	29 (0.4)
Missing/no response	77	529
**Aboriginal and Torres Strait Islander origin**		
Neither Aboriginal nor	21717 (98.7)	8402 (99.5)
Torres Strait Islander origin		
Aboriginal origin	254 (1.1)	41 (0.5)
Torres Strait Islander origin	24 (0.1)	3 (0.04)
Both Aboriginal and	18 (0.1)	1 (0.01)
Torres Strait Islander origin		
Missing/ no response	113	62
**Language other than English spoken at home**		
Yes	7177 (32.5)	*Not collected*
No	14884 (67.5)	
Missing/ no response	65	

Percentages are expressed as percentage of valid responses to each question (ie excluding missing responses).

*Gender was asked in the Commencing Questionnaires, but was not asked specifically in the Exit Questionnaires – results tabulated for Exit Questionnaire responses were derived from responses from the same students for the Commencing Questionnaires, from student names where possible, or from Medical Schools data collections where possible.

Just over half of commencing medical students were under 20 years of age (52.5%, Table 3). The great majority were Australian citizens, and were of neither Aboriginal nor Torres Strait Island origin (Table 3). Nearly one third (32.5%) of commencing medical students spoke a language other than English at home (Table 3).

#### Completing medical students

At completion of medical school, 55% of respondents in the responding cohorts were female. Nearly half (47.8%) of respondents completing Exit Questionnaires were aged 25 to 29 years (Table 3). Just over 80% were Australian citizens. Only 0.55% reported that they were of Aboriginal and/or Torres Straits Islander origin.

### Vocational practice intentions

Students were asked to select, from standardised lists, their first preference for future vocational practice in both the commencing and exit questionnaires. Results are presented in Table 4. Of 22,126 commencing student respondents, 4,791 did not choose any response option. A further 1,684 students chose the ‘not yet decided’ option available in the 2005, 2006 and 2007 questionnaires (this option was not available in questionnaires after 2007). Therefore 15,651 commencing students (70.7%) selected a vocation as their first preference for future practice. Of those nominating a vocational practice first preference, the three highest ranking vocations for commencing students were Surgery (26.4%), Paediatrics and Child Health (15.3%), and General Practice (13.3%) (Table 4).

**Table 4:** **First preference vocational practice intentions**

	CMSQ	EQ
	N = 22,126 (%)	N = 8509 (%)
**Adult Medicine/Internal Medicine/Physician**	1562 (10.0)	1357 (18.0)
**Anaesthesia**	408 (2.6)	633 (8.4)
**Dermatology**	377 (2.4)	128 (1.7)
**Emergency Medicine**	1282 (8.2)	617 (8.2)
**General Practice**	2081 (13.3)	1003 (13.3)
**Intensive Care Medicine**	183 (1.2)	188 (2.5)
**Medical Administration**	56 (0.4)	20 (0.3)
**Non-Specialist Hospital Practice**	52 (0.3)	7 (0.1)
**Obstetrics and Gynaecology**	886 (5.7)	488 (6.5)
**Occupational Medicine**	8 (0.1)	7 (0.1)
**Ophthalmology**	397 (2.5)	187 (2.5)
**Paediatrics and Child Health**	2402 (15.3)	717 (9.5)
**Pathology**	289 (1.8)	66 (0.8)
**Psychiatry**	507 (3.2)	180 (2.4)
**Public Health Medicine**	149 (1.0)	29 (0.4)
**Radiology**	273 (1.7)	141 (1.9)
**Rehabilitation Medicine**	63 (0.4)	15 (0.2)
**Surgery**	4127 (26.4)	1413 (18.8)
**Other**	549 (3.5)	335 (4.4)
**Missing/no response**	6475*	978

Percentages are expressed as percentage of valid responses (i.e. excluding missing responses).

*Includes 1,684 responding ‘not yet decided’ in the 2005, 2006, 2007 commencing questionnaires, after which the ‘not yet decided’ option was removed from subsequent questionnaires as a response option.

Of 8,509 completing students, 978 did not select any response (there was no ‘not yet decided’ option in any Exit Questionnaire). Therefore 7,531 completing students (88.5%) selected a vocation as their first preference for future practice. The three highest ranking vocations were Surgery (18.8%), Adult Medicine/Internal Medicine/Physician (18.0%), and General Practice (13.3%) (Table 4). The Health Workforce 2025 report indicates that General Practice will be in undersupply [17].

Between CMSQ and EQ, 75.7% of medical students changed their first vocational preference. MSOD has the ability to track and identify the factors that influence career intentions and enables assessment of whether current intentions are turning into behavior.

### Preferred geographic location of future medical practice

The preferred geographic location for future practice for respondents to the Commencing and Exit Questionnaires is shown in Table 5. Overall, students expressed a strong preference for Capital City and Major Urban centre locations for their future medical practice compared with regional city/large town, smaller town, and small communities (Table 5). This preference was more marked for students completing their medical studies. However, a number of analyses on MSOD participants provide evidence that a rural background and exposure to rural placements are associated with a shift towards rural practice intentions [18, 19]. At CMSQ the number of students responding that they did not intend to work in Australia was 7.6%, compared with only 1.6% at EQ.

**Table 5:** **Preferred geographic location of future medical practice**

	CMSQ	EQ
	N = 22,126 (%)	N = 8509 (%)
**Capital City**	12973 (61.9)	5405 (67.6)
**Major Urban centre (>100,000)**	2383 (11.4)	1202 (15.0)
**Regional city or large town (25,000 – 100,000)**	2316 (11.0)	898 (11.2)
**Smaller town (10,000 – 24,999)**	1060 (5.0)	281 (3.5)
**Small community (<10,000)**	651 (3.1)	87 (1.1)
**Not intending to work in Australia Missing**	1586 (7.6)	130 (1.6)
	1157	506

Percentages are expressed as percentage of valid responses (i.e. excluding missing responses).

Longitudinal analyses, and changes with progression in medical school and during training, will form part of future manuscripts.

## Discussion

There have been relatively few comprehensive studies which have been longitudinal encompassing the national continuum of medical school education, early postgraduate experience (internship or pre-registration), and vocational training. In particular, some of the key medical workforce studies [9–13] provide only a ‘snapshot’ view of various programs but are seldom as effective in assessing long-term outcomes. At the time of this report, the MSOD project already has recruited over 30,635 responses from Australian medical students and trainees who are being surveyed prospectively (Table 2), making the project one the largest longitudinally tracked cohorts of medical students in any single research project globally.

The MSOD project is unique with its comprehensive national survey methodology, encompassing medical schools in Australia, and New Zealand. Due to the varying lengths of medical school programs, it will be 2015 before students from all medical schools in both countries will complete the PGY3 questionnaires. By 2021 students who have graduated from all medical schools in both countries will be completing PGY8 questionnaires, and many will have completed their vocational (specialist) training by that time. By the time of implementation of PGY8 questionnaires for all graduates from Australian and New Zealand medical schools, it is anticipated that approximately 24,000 students and trainees will be eligible to complete questionnaires each year.

The data obtained will enable longitudinal comparisons of medical student career intentions with training progression for individual medical student cohorts. As the project continues, it will also be possible to examine trends or differences that may occur in vocational choices for more recent commencing student cohorts in comparison with earlier cohorts. Analyses will be possible which examine differences within cohorts according to factors such as gender, medical school course characteristics such as direct undergraduate entry to medical school or postgraduate entry, self-analysis of rural or urban background, and participants self-rating of nominated career choice influencing factors. This Project is primarily limited by the fact that the questionnaire data gathered is by self report and is not validated by any other sources apart from general medical school curriculum delivery databases.

### Future directions

The strengths of the MSOD are the use of a minimum dataset approach, current high response rates, and development of a database as a national resource that is now well established and is beginning to generate significant research outputs [20].

MSOD faces a number of ongoing challenges including the continued substantial engagement of all Australasian Medical Schools and key stakeholders, the maintenance of high response and retention rates through postgraduate surveys over time; and ongoing resources to sustain the Project. There is an ongoing need to provide contemporary policy advice and research outputs to address some of the current problems being encountered as reform gathers momentum in medical education and workforce development, during the period when the long term information from the cohorts is not fully gathered. All Medical Deans in Australia and New Zealand are strongly committed to the sustainability of this crucial planning and policy resource. A next key step in Australia is formalising the link between MSOD and the Australian Health Professional Registration Authority (AHPRA). Through this collaboration, efficient linkages between the MSOD database and the National Health Workforce dataset, with appropriate privacy constraints, will allow consequent analyses of key educational and training experiences throughout medical student training that influences long term career choices. This will assist earlier detection of vocational workforce trends, and in particular specialties which are undersubscribed and for which there is a major workforce need both in numbers and in geographic location of practice.

An important further priority for Medical Deans Inc. is identification of the possible educational levers and opportunities for exposure that will lead to more medical graduates choosing semi-rural, rural and remote practice for their future medical careers [21]. This workforce maldistribution issue continues to be one of the main policy conundrums facing Australia [22].

Health service delivery occurs in a complex and evolving environment, and attempting to plan for future workforce needs is fraught with difficulties. However it is no longer appropriate to leave overall national planning to traditional market economics of supply and demand. The MSOD project provides a framework for scenario based modelling linking student career intentions with current gaps in the medical workforce [17, 20]. This modelling will then allow long term horizon scanning regarding vocational and skills gaps.

## Conclusions

The MSOD project is already of great value to medical leaders and policymakers. Workforce reform is crucial to the creation of a sustainable health care system for the future. The MSOD project as a national resource will be a key tool as workforce planning reforms are implemented.
